# Decompression of the greater occipital nerve improves outcome in patients with chronic headache and neck pain — a retrospective cohort study

**DOI:** 10.1007/s00701-021-04913-0

**Published:** 2021-07-01

**Authors:** Anja Eskilsson, Eva Ageberg, Hans Ericson, Niklas Marklund, Leif Anderberg

**Affiliations:** 1 Department of Clinical Sciences Lund, Neurosurgery, Lund University, 221 85 Lund, Sweden; 2 Department of Health Sciences, Lund University, Lund, Sweden; 3 Neuroscience, Neurosurgery, Uppsala University, Uppsala, Sweden; 4 Skane University Hospital, Department of Clinical Sciences Lund, Neurosurgery, Lund University, Lund, Sweden

**Keywords:** Surgery, Headache, Greater occipital nerve, Decompression, Neuralgia

## Abstract

**Background:**

Compression of the greater occipital nerve (GON) may contribute to chronic headache, neck pain, and migraine in a subset of patients. We aimed to evaluate whether GON decompression could reduce pain and improve quality of life in patients with occipital neuralgia and chronic headache and neck pain.

**Methods:**

In this retrospective cohort study, selected patients with neck pain and headache referred to a single neurosurgical center were analyzed. Patients (n = 22) with suspected GON neuralgia based on nerve block or clinical criteria were included. All patients presented with occipital pain spreading frontally and to the neck in various degree. Surgical decompression was performed under local anesthesia. Follow-up was made by an assessor not involved in the treatment of the patients, by telephone 2–5 years after the surgical procedure and an interview protocol was used to collect information. The data from the follow-up protocols were then analyzed and reported.

**Results:**

When analyzing the follow-up protocols, decreased headache/migraine was reported in 77% and neck pain was reduced in 55% of the patients.

**Conclusions:**

Decompression of GON(s) may reduce neck pain and headache in selected patients with persistent headache, neck pain, and clinical signs of GON neuralgia. Based on the limitations of the present retrospective study, the results should be considered with caution.

## Introduction

Neck pain is a common symptom with an approximate overall prevalence of 0.4–86.8% (mean: 23,1%) in the general population [17] that often coexists with headache [2]. In a subset of patients, cervical pathology is considered the cause of the headache. Cervicogenic headache is classified as a secondary headache according to International Classification of Headache Disorders**,** third edition (ICHD-3) (2018) of the International Headache Society (IHS) [16], and is estimated to contribute to approximately 18% of all headaches [10].

Different studies suggest that headache may originate from the cervical spine. Mechanical needle stimulation of specific cervical roots can produce pain referred to the head [28]. Diagnostic anesthetic blocks and surgical treatment with nerve root decompression can, in patients with cervical radiculopathy, significantly reduce the headache both from upper (C1–C3) and lower cervical spine (C4–C7) [3, 27]. However, headache from the cervical spine could be difficult to discriminate from common headache syndromes, such as migraine and tension-type headache.

Entrapment and compression of the greater occipital nerve (GON) can cause pain in the posterior part of the head, including the upper part of the cervical spine and migraine. Occipital nerve block is commonly used for the diagnosis and treatment of occipital neuralgia and primary headache conditions, as well as headache after head trauma [11, 30]. Pain produced by GON entrapment and/or compression can be referred to the frontal and periorbital regions via the trigeminocervical complex. This complex has afferents from the face and meninges via the trigeminal nerve, connecting with afferents from the posterior parts of the head via the occipital nerves emerging from the upper three cervical segments. These neural connections may be the substrate for referred pain from the upper cervical levels to the head and the face in degenerative cervical spine disorders as well as after whiplash injury [2, 6].

Surgical decompression of the GON has been used to treat GON neuralgia [9], migraine [7, 13, 14] and headache after whiplash injury [21]. However, it has not been reported whether decompression of the greater occipital nerve(s) also could be a treatment option for headache in patients with concomitant chronic neck pain. Therefore, we retrospectively evaluated a group of patients with headache and neck pain who was operated by decompression of the GON(s). The aim of the study was to evaluate the long-term effects of GON decompression on the patients’ headache and neck-pain.

## Methods and materials

### Participants

All consecutive patients referred to a single neurosurgical unit (the Department of Neurosurgery, Skåne University Hospital, Sweden) due to chronic neck-pain and headache between 2008 and 2012 and subjected to GON decompression were recruited to the present retrospective cohort study. The Regional Ethical Review Board in Lund, Sweden approved the study (Dnr 2016/324). All patients in the present study had undergone surgical decompression of the greater occipital nerve (GON) with the aim of reducing their symptoms. The follow-up was based on a telephone interview using a questionnaire and performed by an interviewer (A.E.) not involved in the treatment of the patients.

Inclusion criteria were longstanding headache and neck pain not responding to conservative treatment (analgesia and treatment by a physiotherapist) and clinical signs of uni- or bilateral neuralgia from the GON, treated with surgical decompression of the clinically symptomatic nerve(s). Exclusion criteria were cervical radiculopathy, other nerve entrapments in the neck and shoulder or other verified related diagnoses explaining the patient’s symptoms. The pre-operative medical records were analyzed in order to identify the most common symptoms reported by the patients to the treating neurosurgeon at the pre-operative visit and a follow-up protocol was designed.

### Diagnosis

All patients were selected for the surgical procedure by an experienced neurosurgeon familiar with the diagnosis and its treatment (L.A.). At time of consultation, a clinical sign of entrapment of the GON(s) led to focused evaluation of this condition. Diagnosis was made by careful history and clinical examination. If palpation and percussion over the nerve caused severe pain in the occipital region and/or caused radiating pain cranially and frontally on the same side as the palpation, the diagnosis of GON entrapment was strongly considered. For differential diagnosis, a diagnostic GON block using local anesthesia (4–5 mL of 1% lidocaine) was used. If this block resulted in a marked relief of neck and headache, GON entrapment was also strongly considered. When other causes for the pain condition were excluded or considered unlikely, the patient was offered decompression of the GON.

### Surgical procedure

The surgery was performed under local anesthesia by a single neurosurgeon (L.A.) and the patient was fully awake during the procedure. The patient was placed in a prone position and a partial shaving of the surgical field was performed. After the area was sterilized, local anesthetics (mepivacaine (Carbocain) 10 mg/mL) were infiltrated in the subcutaneous tissue over the pain-producing nerve branch/es. A vertical incision of approximately 5 cm was made, and the nerve was identified by soft tissue dissection, released from constraining soft tissue and followed through the fascia and muscles. Microscope was used when needed, although not as a standard procedure.

The nerve was released until it was considered to have a free passage. The wound was closed with resorbable subcutaneous sutures in two layers and nylon sutures in the skin. The duration of a unilateral surgical procedure was approximately 30 min. The patients were informed about wound tightness the first days after surgery, the risk of complications such as wound infections and potential loss or reduced sensation in the skin. The stitches were removed in a primary care unit after 12 days.

### Assessment

**Fig. 1:**
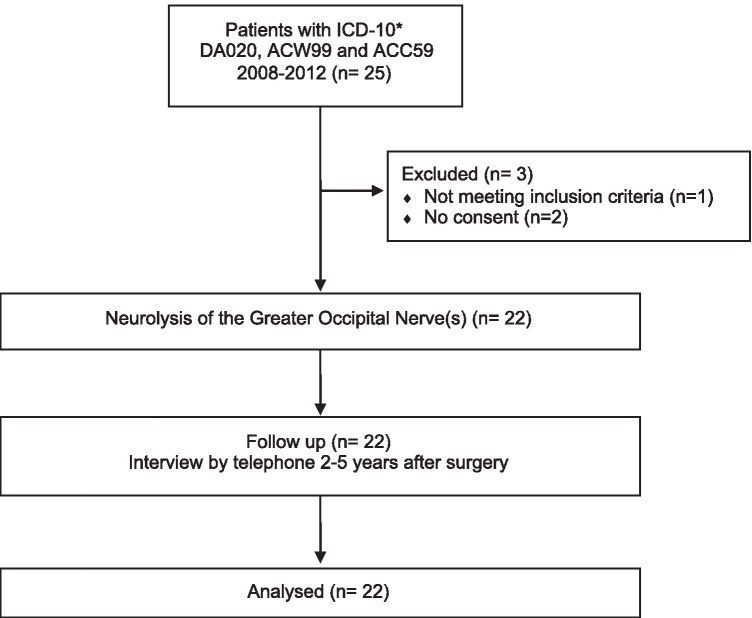
Flow chart of patients included in the present study. * ICD-10: International Classification of Diseases

Follow-up was performed at 2–5 years after surgery by an independent physiotherapist (A.E.) who had not been involved in the care or previous evaluation of the patients. Written information was sent home to the patients, and all patients signed an informed consent. The patients were then interviewed by telephone (Fig. 1). The interviewer used a questionnaire designed to measure symptom changes after transforaminal steroid injections previously used in patients with cervical radiculopathy [1]. The questionnaire was modified to also evaluate symptoms related to occipital neuralgia and factors that may affect quality of life, such as ability to concentrate and read, social and physical activities and ability to work.

### Statistics

For the statistical analysis IBM SPSS Statistics version 22 for Mac was used (IBM Corp., Armonk, NY, USA). Age is presented as mean and standard deviations (SD) and range, for all other data descriptive statistical analysis was used.

## Results

### Patient population

Nine men and 13 women with a mean age of 47 years (range 21–74) fulfilled the inclusion criteria and were enrolled in the present study (Table 1). All patients presented with a clinical picture of symptoms from the GON on one or both sides as their main problem. The GON symptoms included, in all patients, occipital pain spreading frontally and to the neck in various degree. In total, 1/22 (5%) of the patients had previous cervical surgery, and 20/22 (90%) had a history of trauma (motor vehicle accidents, fall injuries, etc.; Table 1).

**Table 1:** Patient characteristics. Characteristics of the included patients undergoing decompression of the greater occipital nerve (GON; n = 22). Age presented as means ± standard deviations and range

Characteristic	
Age (years)	47 ± 14 range (21–74)
Sex (F/M)	n = 13/9
Symptoms*	
Headache	n = 7
Migraine	n = 15
Neck pain	n = 22
Previous cervical surgery	n = 1
Trauma**	n = 20
Diagnostic assessment	
Palpation over GON gives local or radiating pain	n = 20
Nerve block	n = 11
Decompression n = 22	
GON right	n = 12
GON left	n = 4
GON bilateral	n = 6

*The dominant symptoms were headache, migraine and neck pain.

**Traffic accidents, fall injuries.

**Fig. 2:**
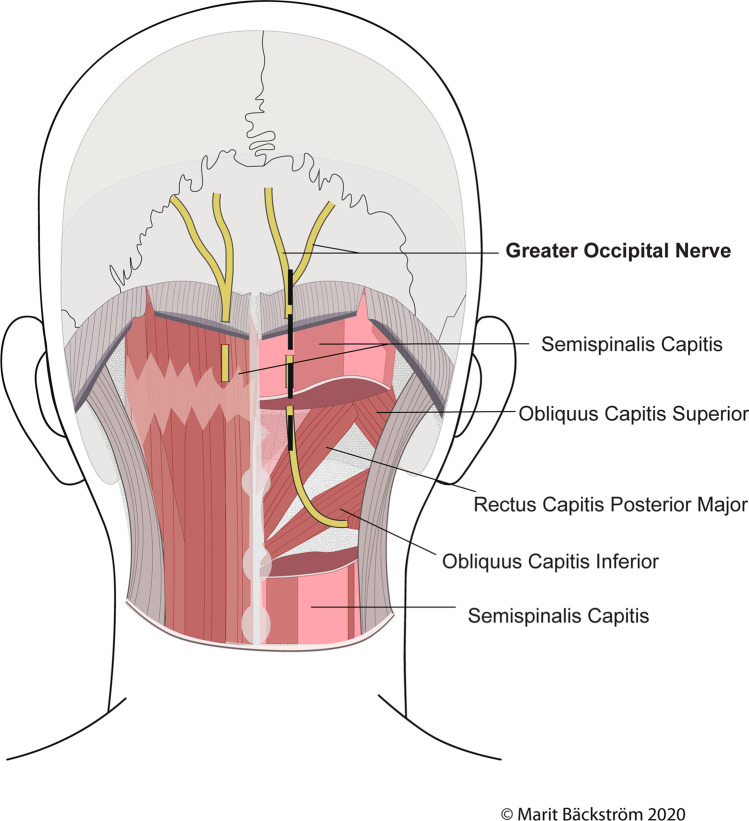
Posterior part of the craniocervical region depicted the bilateral greater occipital nerves. On the right side, the vertical incision for locating the nerve is indicated

At surgery, the course of the nerve varied substantially where some patients had a vertical while others a 45° angle course. Scar tissue was commonly adhering the nerve to surrounding connective tissue, making decompression challenging and nerve branches were occasionally adhered to the scalp connective tissue distaly in the wound. Commonly, the nerve had areas with color alterations. At the GON exit through the semispinalis muscle in the midline, the nerve deviated almost 90° (see Fig. 2 for description of the surgical approach). The area of sharp angulation was also an area of potential nerve compression.

### Surgical outcome

Surgical complications (wound infection, sensory loss) were negligible. However some patients reported deterioration of the pre-surgical, baseline symptoms (Tables 2, 3, 4).

**Table 2:** Follow-up questionnaire — pain. The number (n) of patients who reported perceived changes in symptoms and function at follow-up compared to pre-operative, baseline symptom. *GON*, greater occipital nerve

	Baseline	Follow-up GON decompression			
		Increased	Unchanged	Decreased	Symptom free
**Headache**					
	**n = 7**	n = 0	n = 1	n = 4	n = 2
**Migraine**					
	**n = 15**	n = 2	n = 2	n = 9	n = 2
**Other**					
Neck pain	n = 22	n = 3	n = 7	n = 9	n = 3
Face pain/numbness	n = 15	n = 2	n = 7	n = 4	n = 2
Shoulder pain	n = 17	n = 3	n = 7	n = 5	n = 2
Arm pain	n = 14	n = 2	n = 6	n = 3	n = 3

**Table 3:** Follow-up questionnaire — other symptoms. The number (n) of patients reporting other symptoms and/or problems before decompression of the greater occipital nerve (baseline) and at follow-up

Symptoms	Baseline	Follow-up GON decompression			
		Impaired	Unchanged	Improved	Symptom free
Neck mobility	n = 20	n = 3	n = 7	n = 8	n = 2
Dizziness/instability	n = 15	n = 7	n = 3	n = 2	n = 3
Concentration	n = 21	n = 3	n = 8	n = 8	n = 2
Ability to read	n = 17	n = 3	n = 5	n = 8	n = 1
Social activities	n = 22	n = 4	n = 7	n = 8	n = 3
Mood	n = 22	n = 3	n = 5	n = 11	n = 3
Ability to perform physical activities	n = 22	n = 3	n = 5	n = 11	n = 3
Physical activity level	n = 22	n = 2	n = 9	n = 8	n = 3
Intake of analgesics	n = 21	n = 13	n = 0	n = 5	n = 3
Quality of sleep	n = 22	n = 0	n = 11	n = 10	n = 1
Overall outcome	n = 20	n = 2	n = 0	n = 18	n = 0

76–100%

**Table 4:** Follow-up questionnaire — work ability. Patients subjected to greater occipital nerve (GON) decompression who reported on their ability to work pre-surgery and at follow-up

Work	Baseline		Increased	Unchanged	Decreased
Ability to work	n = 16		n = 7	n = 6	n = 3
**Working capacity %**		**0%**	**25–50%**	**51–75%**	**76–100%**
Prior to GON decompression	n = 22	n = 14	n = 2	n = 0	n = 6
Post GON decompression	n = 22	n = 10	n = 4	n = 1	n = 7

The results from the follow-up questionnaire are given in detail in Tables 2, 3, and 4. In total, 18/20 of the patients reported an overall improvement by surgery. 4/7 of patients reported decreased headache and 2/7 no headache at follow-up (Table 2). Eleven patients reported reduced migraine (Table 2). Neck pain was reduced in 12/22 of the patients. Patient-reported neck mobility was increased in 10/20 of the patients and dizziness/perceived instability was reported reduced in 5/15 of the patients (Table 3). The ability to read text was improved in 9/17 of the patients. In addition, 11/22 of the patients reported better ability to engage in social activities, 14/22 had improved mood and 11/22 reported increased physical ability (Table 3).

### Analgesics and ability to work

Intake of analgesics was decreased in 8/21 of the patients (Table 3), and the work capacity increased from 36 to 54% (Table 4). Frequency of sick-leave of the patient in working age was reduced from 64 to 45% (Table 4).

## Discussion

The present retrospective study describes a cohort of patients with chronic headache, neck-pain and a clinical diagnosis of neuralgia from the greater occipital nerve/s. The patients were clinically investigated by an experienced neurosurgeon who also diagnosed their occipital neuralgia. All patients presented with symptoms from the GON with occipital pain spreading over the skull and to the neck in various degree. This pain was reported as headache and/or migraine and neck-pain from the patients. In this paper, we only use the patients’ own description of their pain (headache or migraine). The same surgeon performed decompression of the symptomatic nerves and two to five years later, the cohort was followed up by a telephone interviewer using a customized protocol. The data from the follow-up protocols were analyzed, and the results presented in this paper, indicate that the surgical decompression reduced the perceived symptoms in this cohort of patients (Table 2, 3).

Interestingly, 20 of the 22 in the cohort had a history of trauma to the head and neck region.

The majority of the patients had a cluster of symptoms difficult to incorporate into one diagnosis as facial pain, arm and shoulder pain coexisted in the cohort but none of the patients had radicular symptoms. However, headache may coexist with cervical radiculopathy [27] and four of our 22 patients were subjected to cervical surgery at some time following GON decompression.

Upper extremity nerve entrapment may cause symptoms such as pain, paresthesia, and muscle weakness [15]. Nerve compression between muscles or other structures in the neck may also affect arm and shoulder strength, because of the involvement of neck muscles in arm and shoulder movements, which illustrates the complexity of the condition. Entrapment and/or compression of the GON can cause neuropathic or neuralgic pain in the posterior part of the head, which can be referred to frontal and periorbital regions ipsilateral to the neuralgia. The anatomy of the GON [18, 25] with its location and lingering course through the posterior cervical muscles makes it vulnerable to compression. The GON may be entrapped in the area where the nerve perforates the aponeurosis of the trapezius muscle [11, 21], in the semispinalis [13, 21] or inferior oblique muscle [12].

We used clinical signs, palpation and GON nerve block using a local anesthetic to establish the surgical indication. Occipital nerve block is commonly used not only for diagnosis but also for the treatment of occipital neuralgia, primary headache conditions, headache after head trauma [11] and whiplash injury [26, 30].

Patients with longstanding pain in the head and neck region often present with secondary symptoms related to the pain. For that reason, our questionnaire was modified to also evaluate these secondary symptoms (Appendix Table 5).

Non-surgical attempts to treat GON entrapment/compression include pharmacological treatment for neuropathic pain and local steroid injections. Occipital nerve stimulation might result in pain relief [22], reduced headache intensity and improved ability to work [23]. In a prospective observational study using electric stimulation of GON in 53 patients with chronic migraine, 40% reported sustained clinical benefit at a mean follow-up of four years [24]. Up to six months delay was reported before clinical effect from the stimulation was achieved. It is difficult to compare the result from this study [24] with our present results since we used different inclusion criteria and patient groups. In a randomized control trial, pulsed radiofrequency (PRF) showed no significant pain relief at follow-up [5]. In contrast, another study found improved daily activity, mood, sleep, and Medication Quantification Scale (MQS) scores at 6 months following treatment [29]. Cryoneurolysis has been attempted in a small series of patients [19]. Surgical decompression of the greater occipital nerve might effectively treat occipital neuralgia [9, 14] and headache after whiplash injury [21]. Using a similar procedure as in our study, Li et al. performed ipsilateral (n = 76) and bilateral (n = 13) micro-surgical decompression of the GON(s) [20] in patients diagnosed with occipital neuralgia. A complete pain relief of the headache symptoms was reported in 90% of the patients at a mean follow-up of 20 months [20]. In that study, patient with previous trauma against the head or neck were not represented, in contrast to the included patients in our present study. The different inclusion criteria in the surgical series investigating treatments of GON neuropathy may affect the outcome, and the interpretation of the results.

Migraine may develop after trauma, and individuals with migraine are also at risk for developing chronic pain following trauma to the head and neck region [16]. Furthermore, the occipital nerves are known to be able to initiate or worsen migraine [4]. Also, occipital neuralgia may lead to referred pain in a facial area related to the trigeminal nerve, symptoms and signs that might be misdiagnosed as migraine. The migraine decreased in 73% of the patients after GON decompression in the present study, indicating functional coexistence of GON neuralgia and migraine. In support of these findings, a previous study using pulsed radiofrequency found that the treatment was more effective on pain relief in patients with occipital neuralgia and migraine than in those without migraine [5, 8]. In a large unselected US population, the self-reported prevalence of migraine during 3 months was 20.7% for women and 9.7% for men [4]. In the present study, the self-reported prevalence of migraine was 68% (15/22).

Shoulder pain and arm pain were also improved in a subset of patients, i.e., 41% and 43%, respectively. One plausible explanation is that in GON compression, arm activity may increase tension and traction of the nerve. Neck pain was a significant problem in all of the patients in our cohort before surgery and was attenuated in > 1/2 of patients at follow-up. The neck pain from the GON might be secondary to increased neck muscle tension or to the neuralgia itself.

While the present study has important strengths such as all surgeries were performed by a single surgeon (L.A.) and that the included patients had all failed prolonged conservative management, there are some important limitations. Since some baseline, i.e. pre-operative, information on patients’ symptoms specified in the questionnaire (concentration, reading, social life, recreation and sleeping) was not available, we cannot exclude potential recall bias at follow-up. Furthermore, this retrospective study is not randomized and decompression was not compared to other treatments modalities. There was also a long period from the surgery to the follow-up, and we cannot exclude that the observed changes were due to the natural course of the condition. However, this explanation can be questioned since patient symptoms were chronic at inclusion and they were non-responders to conservative treatments before undergoing surgical GON decompression. Moreover, although an overall improvement was observed in the majority of patients, there were patients who reported more symptoms at follow-up.

## Conclusions

In selected patients with longstanding headache/migraine and neck pain, presenting with clinical signs of neuralgia from the greater occipital nerve(s), decompression of the nerve(s) appears to reduce patient-reported headache/migraine and neck-pain. Trauma to the head and neck region might precede the clinical condition. Due to the retrospective study design and the potential for recall bias the results have to be considered with care. Prospective studies might overcome these limitations.

## Appendix

**Table 5:** Follow-up protocol

HEADACHE				
Has your headache changed?	No	Increased	Decreased	No Complaints
Did you have migraine before surgery?	No	Yes		
Has your migraine changed?	No	Increased	Decreased	No Complaints
PAIN INTENSITY				
Has your facial pain changed?	No	Increased	Decreased	No Complaints
Has your neck pain changed?	No	Increased	Decreased	No Complaints
Has your shoulder pain changed?	No	Increased	Decreased	No Complaints
Has the pain in your arm(s) changed?	No	Increased	Decreased	No Complaints
SYMPTOMS				
Has the strength in your arm/hand changed?	No	Increased	Decreased	No Complaints
Has your sensibility in your arm/hand changed?	No	Increased	Decreased	No Complaints
Has your mobility in the neck changed?	No	Increased	Decreased	Full Mobility
Has your mobility in the arm/shoulder changed?	No	Increased	Decreased	Full Mobility
How has your dizziness/instability changed?	No	Increased	Decreased	No Complaints
CONCENTRATION				
Has your ability to concentrate changed?	No	Better	Worse	No Complaints
READING				
Has your ability to read changed?	No	Better	Worse	No Complaints
SOCIAL LIFE				
Has your social life changed?	No	Better	Worse	No Complaints
Has your mood changed?	No	Better	Worse	No Complaints
RECREATION				
How has your general performance changed?	No	Better	Worse	No Complaints
Has your physical ability level changed?	No	Increased	Decreased	No Complaints
Have you changed your intake of medication?	No	Increased	Decreased	No Medications
SLEEPING				
Has your night sleep changed?	No	Yes		
WORK				
Has your capacity to work changed?	No	Increased	Decreased	
Has you got customized/new duties?	No	Yes	New work	
How many % did you work before surgery?	0	25	75	100
How many % did you work after surgery?	0	25	75	100
Do you experience any changes (in general) after surgery?	No	Better	Worse	
